# Supplementary material for: Saccharomyces boulardii (CNCM I-745) improves intestinal damage in sepsis by remodeling intestinal flora structure
Source: Front Cell Infect Microbiol. 2025 Jul 31;15:1602792. doi: 10.3389/fcimb.2025.1602792 (PMC12350329; doi:10.3389/fcimb.2025.1602792)
Supplement: Supplementary file 1 [file DataSheet1.zip › summary/summary/6_Taxonomy_community/1_Taxonomy_profiling/3_Class/All/Class_abund_top30.sample.cluster.pdf]

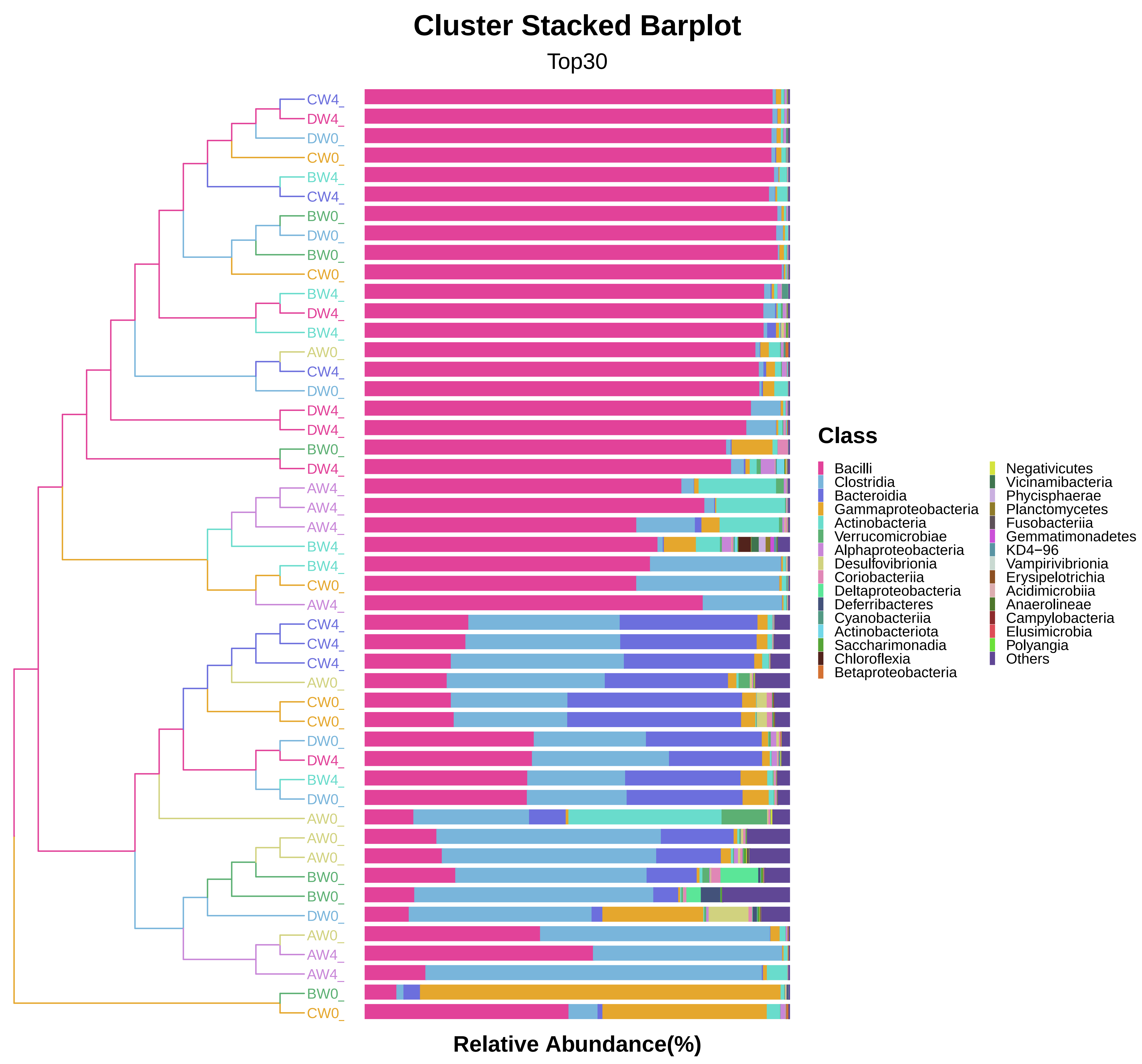
Cluster Stacked Barplot
Top30
CW4_
DW4_
DW0_
CW0_
BW4_
CW4_
BW0_
DW0_
BW0_
CW0_
BW4_
DW4_
BW4_
AW0_
CW4_
DW0_
DW4_
DW4_
BW0_
DW4_
AW4_
AW4_
AW4_
BW4_
BW4_
CW0_
AW4_
CW4_
CW4_
CW4_
AW0_
CW0_
CW0_
DW0_
DW4_
BW4_
DW0_
AW0_
AW0_
AW0_
BW0_
BW0_
DW0_
AW0_
AW4_
AW4_
BW0_
CW0_
Relative Abundance(%)
Class
Bacilli
Clostridia
Bacteroidia
Gammaproteobacteria
Actinobacteria
Verrucomicrobiae
Alphaproteobacteria
Desulfovibrionia
Coriobacteriia
Deltaproteobacteria
Deferribacteres
Cyanobacteriia
Actinobacteriota
Saccharimonadia
Chloroflexia
Betaproteobacteria
Negativicutes
Vicinamibacteria
Phycisphaerae
Planctomycetes
Fusobacteriia
Gemmatimonadetes
KD4−96
Vampirivibrionia
Erysipelotrichia
Acidimicrobiia
Anaerolineae
Campylobacteria
Elusimicrobia
Polyangia
Others
